# Mutations that permit residual CFTR function delay acquisition of multiple respiratory pathogens in CF patients

**DOI:** 10.1186/1465-9921-11-140

**Published:** 2010-10-08

**Authors:** Deanna M Green, Kathryn E McDougal, Scott M Blackman, Patrick R Sosnay, Lindsay B Henderson, Kathleen M Naughton, J Michael Collaco, Garry R Cutting

**Affiliations:** 1 Division of Pediatric Respiratory Sciences, Johns Hopkins University School of Medicine, Baltimore, MD, USA; 2 Department of Epidemiology, Bloomberg School of Public Health, Johns Hopkins University, Baltimore, MD, USA; 3 McKusick-Nathans Institute of Genetic Medicine, Johns Hopkins University School of Medicine, Baltimore, MD, USA; 4 Pediatric Endocrinology, Johns Hopkins University School of Medicine, Baltimore, MD, USA

## Abstract

**Background:**

Lung infection by various organisms is a characteristic feature of cystic fibrosis (CF). *CFTR *genotype effects acquisition of *Pseudomonas aeruginosa (Pa)*, however the effect on acquisition of other infectious organisms that frequently precede *Pa *is relatively unknown. Understanding the role of CFTR in the acquisition of organisms first detected in patients may help guide symptomatic and molecular-based treatment for CF.

**Methods:**

Lung infection, defined as a single positive respiratory tract culture, was assessed for 13 organisms in 1,381 individuals with CF. Subjects were divided by predicted CFTR function: 'Residual': carrying at least one partial function *CFTR *mutation (class IV or V) and 'Minimal' those who do not carry a partial function mutation. Kaplan-Meier estimates were created to assess *CFTR *effect on age of acquisition for each organism. Cox proportional hazard models were performed to control for possible cofactors. A separate Cox regression was used to determine whether defining infection with *Pa*, mucoid *Pa *or *Aspergillus (Asp) *using alternative criteria affected the results. The influence of severity of lung disease at the time of acquisition was evaluated using stratified Cox regression methods by lung disease categories.

**Results:**

Subjects with 'Minimal' CFTR function had a higher hazard than patients with 'Residual' function for acquisition of 9 of 13 organisms studied (HR ranging from 1.7 to 3.78 based on the organism studied). Subjects with minimal CFTR function acquired infection at a younger age than those with residual function for 12 of 13 organisms (p-values ranging: < 0.001 to 0.017). Minimal CFTR function also associated with younger age of infection when 3 alternative definitions of infection with *Pa*, mucoid *Pa *or *Asp *were employed. Risk of infection is correlated with CFTR function for 8 of 9 organisms in patients with good lung function (>90%ile) but only 1 of 9 organisms in those with poorer lung function (<50%ile).

**Conclusions:**

Residual CFTR function correlates with later onset of respiratory tract infection by a wide spectrum of organisms frequently cultured from CF patients. The protective effect conferred by residual CFTR function is diminished in CF patients with more advanced lung disease.

## Background

Cystic fibrosis (CF) is the most common autosomal recessive life-shortening disorder in the Caucasian population and progressive obstructive lung disease is the primary cause of mortality[1,2]. Persistent respiratory infection by particular organisms, such as *Pseudomonas aeruginosa *(*Pa*) and *Burkholderia cepacia *complex (*Bcc*), are hallmarks of CF lung disease and lead to the progressive obstructive lung disease[2-5]. Infection with *Pa *is often preceded by infection with other organisms that are rarely cultured from healthy individuals such as *Staphylococcus aureus*. Additionally, a number of other pathogens such as methicillin resistant *Staphylococcus aureus *(MRSA)[6], *Achromobacter xylosoxidans *(*Ax*)[7] and *Aspergillus fumigatus *(*Asp*)[8] have been found to infect CF patients and affect the severity of lung disease in these patients. Mutations which permit residual *CFTR *function and confer a "mild" phenotypic effect have been associated with lower rates of *Pa *infection[9,10]. However, it is not clear whether residual *CFTR *function also influences acquisition of respiratory pathogens other than *Pa *[11].

Teasing out the role that *CFTR *genotype has on CF lung infection is important for several reasons. Recent success in the identification of small molecules augmenting CFTR function raises the possibility that delay of infection might be used as a measure for clinical efficacy of these new treatments, especially in children where few alternative options exist[12]. Secondly, patient populations have been accrued to find genetic modifiers of traits such as risk of infection and quantifying the contribution *CFTR *has to infection aids the search for genetic modifiers of this trait providing novel therapeutic targets for CF. Finally, neonatal CF pigs show acquisition of infection with a variety of organisms indicating that loss of CFTR function causes a pervasive defect in lung defense[13]. If this is the case in humans, then alteration in CFTR function should correlate with infection with a variety of respiratory pathogens. To address these issues, we evaluated the correlation between *CFTR *genotype and infection with a variety of pathogens using detailed infection data collected by the U.S. CF Twin and Sibling Study (CFTSS)[14].

## Methods

### Population

All subjects in the U.S. CFTSS were recruited on the basis of having a twin or sibling affected with CF. All enrolled patients met CF diagnostic criteria[15] and 99% attend CF care centers in the United States. Isolation of patient DNA, identification of *CFTR *mutations, and zygosity testing have been previously described[14,16]. Written, informed consent/assent was obtained from all subjects and/or their guardians, and this study was approved by the Johns Hopkins University Institutional Review Board.

### Definitions of infection

Thirteen organisms were studied including *Pseudomonas aeruginosa (Pa)*, mucoid *Pa *(M*Pa*), *Staphylococcus aureus *(*Sa*), Methicillin resistant *Sa *(MR*Sa*), *Burkholderia cepacia *complex (*Bcc*), Atypical mycobacteria (atyp), *Aspergillus fumigatus *(*Asp*), *Stenotrophomonas maltophilia *(*Sm*), *Achromobacter xylosoxidans *(*Ax*), *Haemophilus influenzae *(*Hi*), *Streptococcus pneumoniae *(*Sp*), *Escherichia coli *(*Ec*), and *Klebsiella pneumoniae *(*Kp*). Subjects were defined as having an "infection" with the organism in question if they had a positive oropharyngeal culture (throat, sputum, or bronchoalveolar lavage culture) for that particular organism (FirstInfx), regardless of whether the subject was positive for other organisms. An additional inclusion criterion was that a negative culture for the organism had to have been obtained a minimum of one week prior to the first positive culture to insure that subjects were in follow-up and routine cultures were being obtained. Patients treated at CF care centers in the U.S. have complete microbiological assessment of respiratory cultures at least annually and preferably quarterly. All subjects were passively followed-up after enrollment in the CFTSS until December 31, 2008 with culture data provided from their care centers and the CF Foundation Patient registry. Subjects were excluded if a respiratory culture was never performed prior to December 31, 2008.

A separate analysis of infection with *Pa*, M*Pa *and *Asp *was performed using 3 additional criteria to define infection: 1) chronic infection (ChronInfx): 3 positive cultures within 6 months with each culture separated by at least 1 month (similar to that employed in Europe)[17] 2) multiple infection (MultiInfx): at least 3 positive cultures, but not meeting the definition for ChronInfx (as most patients in the U.S. do not attend CF clinic 3 times in 6 months); and 3) persistently infected (PersInfx): multiple cultures obtained in 3 consecutive years with positive cultures observed in at least 2 of the 3 years (recently used in a CF modifier study[18]).

### *CFTR *genotypes and other variables

Subjects were grouped according to the functional class of *CFTR *mutations that he/she carried[10,19-22] (TABLE 1). Lung function variables were created using the maximum FEV_1 _within the year preceding the date of the first positive culture for a given organism referenced against the CF population (Kulich CF specific FEV_1 _percentile)[23] (FEV_1_BeforeInfx). The number of cultures per year for the subject was determined as the number of respiratory cultures obtained from the subject divided by the time (in years) from first documented culture to the last recorded culture (cultures per year). Age of acquisition was defined as the time from birth until recorded first positive culture for a given organism. Subjects who did not have a positive culture for the organism were assigned their age at the last obtained culture.

**Table 1:** Classification of CFTR alleles

Category	Mutation	Specific mutations
**Class I**	Defective Protein Synthesis (nonsense, frameshift, aberrant splicing)	1078delT, 1154 insTC, 1525-2A > G, 1717-1G > A, 1898+1G > A, 2184delA, 2184 insA, 3007delG, 3120+1G > A, 3659delC, 3876delA, 3905insT, 394delTT, 4010del4, 4016insT, 4326delTC, 4374+1G > T, 441delA, 556delA, 621+1G > T, 621-1G > T, 711+1G > T, 875+1G > C, E1104X, E585X, E60X, E822X, G542X, G551D/R553X, Q493X, Q552X, Q814X, R1066C, R1162X, R553X, V520F, W1282X, Y1092X

**Class II**	Abnormal Processing and Trafficking	A559T, D979A, ΔF508, ΔI507, G480C, G85E, N1303K, S549I, S549N, S549R

**Class III**	Defective Channel Regulation/Gating	G1244E, G1349D, G551D, G551S, G85E, H199R, I1072T, I48T, L1077P, R560T, S1255P, S549(R75Q)

**Class IV**	Decreased Channel Conductance	A800G, D1152H, D1154G, D614G, delM1140, E822K, G314E, G576A, G622D, G85E, H620Q, I1139V, I1234V, L1335P, M1137V, P67L, R117C, R117P, R117H, R334W, R347H, R347P, R347P/R347H, R792G, S1251N, V232D

**Class V**	Reduced Synthesis and/or Trafficking	2789+5G > A, 3120G > A, 3272-26A > G, 3849+10kbC > T, 5T variant, 621+3A > G, 711+3A > G, A445E, A455E, IVS8 poly T, P574H

### Statistical analysis and regression modeling

Time to acquisition of the first positive culture for an organism was assessed by the Kaplan Meier product limit estimator and the log-rank test. Cox proportional hazard regression models were used to account for covariates which could affect the relationship of *CFTR *genotype and age of acquisition for a particular organism. Covariates were identified a priori based on clinical experience and review of literature. Univariate regression was first performed with co-variates of gender (male vs. female), ethnicity (Caucasian vs. non-Caucasian), pancreatic status (pancreatic insufficient vs. pancreatic sufficient), FEV_1_BeforeInfx (linear co-variate), and cultures per year (linear co-variate) to identify significant co-variates. Co-variates with a p-value < 0.05 (pancreatic status, FEV_1_BeforeInfx, and cultures per year) were included in a multivariate Cox regression. In this regression, only FEV_1_BeforeInfx and cultures per year remained significant with p-values < 0.05. The final multivariate Cox regression model included FEV_1_BeforeInfx for the organism and cultures per year.

A second analysis was performed also using Cox regression to assess the relationship of *CFTR *with alternative ways of defining infection status for *Pa*, M*Pa *and *Asp*. This regression was also adjusted for FEV_1_BeforeInfx for the organism and cultures per year. The time to "event" was defined as the age of the subject from birth until the first positive culture that would be included to meet the infection definition. In other words, for a subject meeting the definition of ChronInfx, the subject would need 3 positive cultures within 6 months but each 1 month from the other and the age of the subject at the first of the 3 positive cultures would be used. If subjects did not meet the definition of infection the age at the last obtained culture is used for censoring. All statistical calculations were performed using Intercooled Stata 10 (Stata Corp., College Station, TX).

## Results

### Clinical characteristics and infection rates in the study population

1,659 CF patients were enrolled in the U.S. CFTSS as of December 31, 2008. Thirty-five subjects were excluded for lack of infection data; 16 for lack of *CFTR *genotype information; 227 subjects for inability to classify *CFTR *mutations. The remaining 1,381 subjects were stratified according to predicted mutation effect upon CFTR function. 'Residual' function was defined as the presence of at least one class IV or V mutation (n = 163) while 'Minimal' function was defined as presence of only class I, II or III mutations (n = 1218). As expected from previous reports[10,11] subjects grouped according to predicted CFTR function differed for multiple characteristics (TABLE 2). Prevalence data for the organisms studied as well as comparability of the study sample to the general U.S. CF population is available in TABLE 3.

**Table 2:** Overall prevalence of organisms in the U.S. CF Twin and Sibling Study, prevalence of infections in the U.S. CF Twin and Sibling Study in 2008 and the U.S. CF Foundation Registry[29] in 2008.

	U.S. CF Twin and Sibling Study Overall	U.S. CF Twin and Sibling Study 2008	CF Foundation Registry 2008
***Pa***	85.4%	46.9%	52.5%

***MPa***	57.5%	Not estimated	Not estimated

***Asp***	40.3%	Not estimated	Not estimated

***Sa***	94.3%	48.1%	50.9%

***MRSa***	39.2%	23.6%	22.6%

***Bcc***	7.8%	2.1%	2.8%

***Atyp***	7.3%	Not estimated	Not estimated

***Sm***	41.6%	11.6%	12.5%

***Ax***	19.6%	Not estimated	Not estimated

***Hi***	74.2%	15.0%	16.3%

***Sp***	35.8%	Not estimated	Not estimated

***Ec***	19.4%	Not estimated	Not estimated

***Kp***	19.8%	Not estimated	Not estimated

Abbreviations: *Pa: Pseudomonas aeruginosa *M*Pa *: mucoid *Pseudomonas aeruginosa*, *Asp*: *Aspergillus fumigatus*, *Sa: Staphylococcus aureus*, MR*Sa*: Methicillin resistant *Staphylococcus aureus*, *Bcc*: *Burkholderia cepacia *complex, atyp: Atypical mycobacteria, *Sm*: *Stenotrophomonas maltophilia*, *Ax: Achromobacter xylosoxidans*, *Hi*: *Haemophilus influenzae*, *Sp: Streptococcus pneumoniae*, *Ec: Escherichia coli *and *Kp*: *Klebsiella pneumoniae*

**Table 3:** Characteristics of study subjects

	'Minimal' Function	'Residual' Function	p value
**Individuals**	1218	163	

**Female**	579 (47.5%)	81 (49.7%)	0.605 ^a^

**Caucasian**	943 (95.5%)	115 (83.9%)	**<0.001**^b^

**Pancreatic Insufficient**	1180 (97.8%)	46 (30.3%)	**<0.001**^b^

**Age (yrs) last visit ± SD (range), (n)**	15.82 ± 8.60 (0.44-57.31) (1209)	18.59 ± 12.87 (0.54-63.94) (159)	0.133^b^

**Maximal FEV**_**1**_**CF% since last clinic visit, (n)**	0.68 ± 0.26 (1111)	0.75 ± 0.25 (145)	**<0.001**^b^

**Average number of cultures per subject per year ± SD, (n)**	3.89 ± 1.92 (1201)	3.93 ± 2.27 (159)	0.530 ^b^

Definition of abbreviations: Maximal FEV_1 _CF%: CF specific percentile for the maximum forced expiratory volume in one second in the last year of data for the subject.

a: P-value reported from chi-squared, assumes equal variance in group means.

b: P-value reported from Kruskal-Wallis test due to unequal variances in group means.

### Residual CFTR function decreases the risk of respiratory infection by multiple organisms

**Figure 1:**
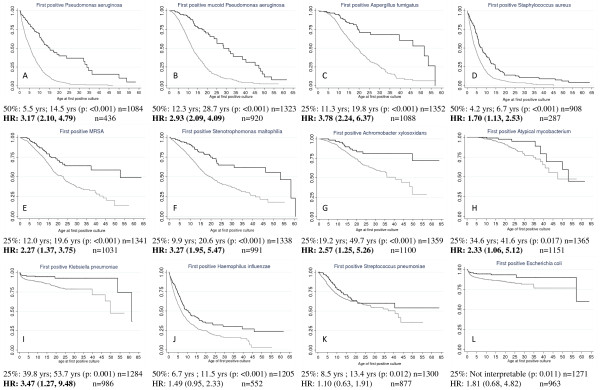
**Unadjusted Kaplan-Meier (K-M) plots for 12 of 13 infectious organisms studied**. Gray lines represent 'Minimal' CFTR function subjects and black lines represent subjects with 'Residual' CFTR function. There are differing numbers of subjects used in each plot due to the inclusion criteria of having a previous negative culture prior to the first positive culture for the particular organism. Median age differences are presented for those infections not affected by censoring, otherwise ages corresponding to the point where 25% of the population is affected is presented. Corresponding t-tests for the unadjusted K-M and Cox proportional hazard ratios with 95% confidence interval adjusted for the FEV_1_BeforeInfx and the number of cultures per subject per year are presented. A. *Pseudomonas aeruginosa*. B. mucoid *Pseudomonas aeruginosa*. C. *Aspergillus fumigatus*. D. *Staphylococcus aureus*. E. Methicillin-resistant *Staphylococcus aureus*. F. *Stenotrophomas maltophilia*. G. *Achromobacter xylosoxidans*. H. Atypical mycobacterium. I. *Klebsiella pneumoniae*. J. *Haemophilus influenzae*. K. *Streptococcus pneumoniae*. L. *Escherichia coli*. *Burkholderia cepacia *complex is not presented as few subjects developed this infection and results were limited in interpretation.

Kaplan Meier analysis was performed to assess the effect of *CFTR *genotype upon the age at which the infection was acquired for 13 organisms. The unadjusted median age of acquisition for *Pa *for subjects with 'Minimal' CFTR function was substantially lower (5.5 years) than those with 'Residual' CFTR function (14.5 years; log rank p-value: < 0.001). Overall prevalence of *Asp *in this study was less than 50%, we report the age at which 25% of the population would be affected. Patients with 'Minimal' function reached 25% prevalence at11.3 years while those with 'Residual' function reached this rate at 19.8 years (p-value < 0.001). Ten other organisms also had a lower age of acquisition in those subjects with 'Minimal' CFTR function when compared to 'Residual' function using log rank tests. (See FIGURE 1 for further details).

As noted in TABLE 2, subjects with 'Residual' CFTR function and those with 'Minimal' CFTR function differ in a number of respects, therefore a Cox proportional hazards model was created that incorporated these difference as covariates. The final multivariate Cox regression included lung function (FEV_1_BeforeInfx) and cultures per year. Nine of the 13 organisms were found to have a significant hazard ratio (HR) indicating an increased risk of acquisition in subjects with 'Minimal' CFTR function (FIGURE 1). For *Pa*, the hazard ratio was increased 3 fold for those with 'Minimal' function when compared to those with 'Residual' function. Increased risk for the other 8 organisms ranged from 1.7 to 3.8. *Bcc *is not presented in the figure as it was found to not be associated with *CFTR *genotype by log rank or Cox regression.

### Residual CFTR function decreases risk of infection with *Pa*, M*Pa *and *Asp *regardless of infection definition

Various criteria have been employed in the literature to define infection status[17]. As this study is retrospective and possibly prone to misclassification, we determined whether the use of different criteria with more rigorous definitions for infection altered the results. Patients were classified for infection status for *Pa*, M*Pa *and *Asp *using three other definitions (ChronInfx, MultiInfx and PersInfx). The number of subjects defined as positive using each definition is presented in TABLE 4. One-hundred nineteen subjects were positive for *Pa *by all 4 methods. Forty-one subjects were positive by 2 of 4 definitions, most commonly meeting the definition of FirstInfx and MultiInfx. Only 55 subjects were positive by the definition for FirstInfx. Similar concordance was found for M*Pa *with 180 subjects positive using all 4 definitions and 78 only positive for FirstInfx. For *Asp*, fewer subjects were positive for all four definitions (n = 76) and 140 were only positive if FirstInfx was used to define infection.

**Table 4:** Risk of infection is increased for 'Minimal' CFTR function using any definition of infection with *Pseudomonas aeruginosa*, mucoid *Pseudomonas aeruginosa *and *Aspergillus fumigatus.*

	Total Positive (n included in analysis)	'Minimal' Function (% of class positive)	'Residual' Function (% of class positive)	**Adjusted Hazards Ratio**^**a**^
**FirstInfx *Pa***	318 (436)	278 (79.4%)	40 (46.5%)	**3.17 (2.10, 4.78)**

**ChronInfx *Pa***	127 (436)	118 (33.7%)	9 (10.5%)	**5.47 (2.20, 13.58)**

**MultiInfx *Pa***	229 (436)	206 (58.9%)	23 (26.7%)	**3.81 (2.32, 6.28)**

**PersInfx *Pa***	228 (436)	203 (58.0%)	25 (29.1%)	**3.32 (2.00, 5.50)**

**FirstInfx M*Pa***	492 (919)	453 (56.3%)	39 (33.9%)	**2.93 (2.09, 4.09)**

**ChronInfx M*Pa***	230 (919)	217 (27.0%)	13 (11.3%)	**3.85 (2.00, 7.39)**

**MultiInfx M*Pa***	381 (919)	354 (44.0%)	27 (23.5%)	**3.11 (2.09, 4.62)**

**PersInfx M*Pa***	371 (919)	345 (42.9%)	26 (22.6%)	**3.14 (2.06, 4.76)**

**FirstInfx Asp**	451 (1090)	427 (44.0%)	24 (20.0%)	**3.77 (2.24, 6.35)**

**ChronInfx Asp**	89 (1090)	88 (9.1%)	1 (0.8%)	**18.97 (3.73, 96.43)**

**MultiInfx Asp**	252 (1090)	237 (24.4%)	15 (12.5%)	**3.22 (1.73, 5.98)**

**PersInfx Asp**	254 (1090)	243 (25.1%)	11 (9.2%)	**4.83 (2.47, 9.46)**

Definition of abbreviations: *Pa*: *Pseudomonas aeruginosa*, M*Pa: *mucoid *Pseudomonas aeruginosa*, Asp: *Aspergillus fumigatus*, FirstInfx: one positive culture for the organism; MultiInfx: three or more positive cultures for the organism; PersInfx: at least one positive culture for the organism in two of three consecutive years; ChronInfx: three positive cultures within 6 months each positive culture at least 1 month from the last positive culture.

a: Adjusted for FEV_1_BeforeInfx and number of cultures.

Multivariate Cox regression was used to assess the relationship of *CFTR *genotype with acquisition of infection using each of these different definitions as the primary outcome. Subjects with 'Minimal' CFTR function had a substantially higher risk of acquiring *Pa*, M*Pa *and *Asp *than those with 'Residual' CFTR function for any definitions used (TABLE 4). Thus, use of an alternate definition of infection does not substantially alter the association between predicted CFTR function and acquisition of *Pa*, M*Pa *or *Asp*.

### CFTR associated risk of infection is influenced by lung function status at the time of acquisition

Lung function affected the relationship of *CFTR *and infection risk and was retained as a significant covariate in all Cox regression models. To better understand this relationship, we performed a formal test of interaction of lung function as a linear covariate and CFTR as a dichotomous variable. The interaction term of FEV_1_BeforeInfx and CFTR was not significant for each organism with the exception of Klebsiella which was found to be highly significant (p-value = 0.008). However, we did not feel that this adequately represented what we observed in our data and therefore wanted to confirm this observation using stratification. Four strata distributed subjects into roughly equal size groups for each infecting organism (FEV_1_BeforeInfx less than the 50^th ^%ile, 50-74.9^th ^%ile, 75-89.9^th ^%ile and greater than the 90^th ^%ile) and revealed that the effect of *CFTR *genotype upon risk of infection differed depending upon lung function and the organism involved (TABLE 5). *CFTR *genotype confers a significant risk to the acquisition of 8 of 9 organisms for patients in the highest strata of lung function (i.e. >90^th ^%ile). However, at the lowest lung function strata (<50^th ^%ile), *CFTR *genotype significantly alters infection risk with only 1 organism (*Sa*). These results suggest that protection from lung infection originally conferred by 'Residual' CFTR function is lost as lung function declines.

**Table 5:** Lung strata^a ^specific hazard ratios for the association of presence of an infection with class mutation status

	**Multivariate regression**^**b**^	**Lung Function <0.50**^**c**^	**Lung Function 0.50-0.75**^**c**^	**Lung Function 0.75-0.9**^**c**^	**Lung Function >0.9**^**c**^
***Pa***	**3.17 (2.10, 4.78)**	1.59 (0.78, 3.24) (n = 81)	**3.74 (1.70, 8.22) (n = 112)**	**4.17 (1.45, 12.0) (n = 110)**	**4.01 (1.92, 8.36) (n = 133)**

**M*Pa***	**2.93 (2.09, 4.09)**	1.59 (0.78, 3.22) (n = 192)	**3.80 (2.07, 6.98) (n = 227)**	**4.23 (2.01, 8.92) (n = 246)**	**3.83 (2.06, 7.11) (n = 255)**

***Asp***	**3.78 (2.24, 6.37)**	2.85 (0.77, 10.63) (n = 250)	**10.22 (3.25, 32.21) (n = 282)**	**4.51 (1.46, 13.92) (n = 269)**	**2.82 (1.34, 5.93) (n = 289)**

***Sa***	**1.70 (1.13, 2.53)**	**3.41 (1.43, 8.16) (n = 74)**	1.05 (0.54, 2.03) (n = 83)	0.90 (0.40, 2.06) (n = 66)	**1.94 (1.02, 3.70) (n = 64)**

**MR*Sa***	**2.27 (1.37, 3.75)**	2.04 (0.71, 5.87) (n = 237)	2.06 (0.84, 5.05) (n = 262)	1.80 (0.76, 4.30) (n = 260)	**3.40 (1.41, 8.21) (n = 273)**

**Atyp**	**2.33 (1.06, 5.12)**	2.12 (0.27, 16.66) (n = 265)	5.41 (0.42, 70.41) (n = 294)	1.40 (0.52, 3.78) (n = 292)	**2.47 (1.06, 5.72) (n = 303)**

***Sm***	**3.27 (1.95, 5.47)**	2.70 (0.88, 8.31) (n = 229)	**3.62 (1.52, 8.62) (n = 257)**	**2.45 (1.12, 5.33) (n = 260)**	**3.64 (1.31, 10.13) (n = 247)**

***Ax***	**2.57 (1.25, 5.26)**	1.94 (0.42, 9.05) (n = 252)	**8.41 (1.83, 38.58) (n = 282)**	2.07 (0.59, 7.30) (n = 275)	1.93 (0.64, 5.82) (n = 293)

***Kp***	**3.47 (1.27, 9.48)**	0.28 (0.07, 1.06) (n = 233)	**7.87 e15 (2.30 e15, 2.69 e16) (n = 250)**	**7.55 e15 (2.60 e15, 2.19 e16) (n = 247)**	**4.73 (1.15, 19.43) (n = 258)**

Definition of abbreviations: *Pa: Pseudomonas aeruginosa *M*Pa *: mucoid *Pseudomonas aeruginosa*, *Asp: Aspergillus fumigatus*, *Sa: Staphylococcus aureus*, MR*Sa*: Methicillin resistant *Staphylococcus aureus*, atyp: Atypical mycobacteria, *Sm*: *Stenotrophomonas maltophilia*, *Ax: Achromobacter xylosoxidans*, *Kp*: *Klebsiella pneumoniae.*

a: Individual subjects may be in different strata for each infection as the value for the lung function variable reflects the maximal value in the year preceding acquisition of each of the organisms.

b: Cox regression adjusted for FEV_1_BeforeInfx and number of cultures per year for the subject; comparing 'Residual' function to 'Minimal' function.

c: Stratified Cox regression adjusted for number of cultures per year for the subject; comparing 'Residual' function to 'Minimal' function in each stratum.

## Discussion

Pulmonary disease in CF is complicated by patients acquiring numerous different organisms at different periods of time during his/her lifetime. These organisms tend to progress from *Sa *and *Hi *to *Pa*. While *Pa *has been extensively studied for its relationship with *CFTR *mutations, the underlying defect of CF, few studies have evaluated the effect that *CFTR *genotype has on infection risk for other organisms commonly cultured from CF respiratory tracts. Our study corroborates the finding that *CFTR *mutations which permit 'Residual" CFTR function (i.e. class IV or V mutations) are associated with reduced rates and delayed acquisition of *Pa *infection[10,11]. We substantially extend this observation to include numerous other organisms commonly cultured from CF patients (mucoid *Pa*, *Staphylococcus aureus*, MRSA, *Aspergillus fumigatus*, *Stenotrophomonas maltophilia*, *Achromobacter xylosoxidans*, atypical mycobacterium and *Klebsiella pneumoniae*) indicating that CFTR dysfunction appears to cause a global alteration in infection resistance in humans. This concept is consistent with recent studies which indicate loss of CFTR function causes a pervasive defect in resistance to respiratory infection in pig models of CF[13]. Additionally this study shows that the concept of "mild" *CFTR *mutations (often 'Residual' function mutations) versus "severe" *CFTR *mutations (often 'Minimal' function mutations) may extend beyond pancreatic status to other features such as lung infection. Furthermore, we show that the association between *CFTR *genotype and respiratory infection has a complex relationship with the severity of lung disease. *CFTR *mutations that permit 'Residual' function reduce the risk of infection with multiple organisms in patients with mild lung dysfunction evidenced by near normal forced expiratory volumes. However, in patients with compromised lung function, the protective effect of 'Residual' CFTR function is lost for most of the organisms studied.

To date, studies on the role of *CFTR *as a risk factor for acquisition of infectious organisms have concentrated on the most common mutation, F508del. A few studies have assessed the role of the *CFTR *genotype beyond the classification of F508del homozygotes versus all others. One study found that mis-sense and splice mutations predicted to confer "mild" effect were associated with lower rates and later acquisition of *Pa *infection[9]. However, mis-sense and splice-site mutations have different effects upon CFTR function; the former being associated with protein formation (and possible residual function) while the latter is usually associated with no protein being made (and no residual function)[24]. Two subsequent studies[10,11] demonstrated that stratifying *CFTR *mutations by putative function was associated with lower rates of *Pa *infection. One of these studies[11] attempted to associate *CFTR *mutation class with infection risk for other organisms but failed to show a reliable association. One reason this current study found an association of *CFTR *genotype with multiple respiratory pathogens may be due to the increased power afforded by a larger sample size when subjects with class IV or V mutations are combined (as opposed to assessing each mutation class separately[11]). Alternatively, non-genetic factors may play a role as the prior study was performed in Europe as opposed to the current U.S. study.

Furthermore, the role of CFTR in the acquisition of lung infection has been debated. One possibility is that CFTR on epithelial cell membranes binds organisms directly by recognizing the lipopolysaccharide coat of *Pa *[25] thus allowing endocytosis and eventual destruction of *Pa*. Loss of this bacterial clearance process due to mutations that do not permit residual CFTR function (class I, II or III) is postulated to underlie the chronic infectious state associated with *Pa*[26]. This however does not fully explain the relationship observed for CFTR and other organisms. A second hypothesis posits that defective clearance of infecting organisms due to altered properties of airway surface liquid and innate defense caused by CFTR dysfunction [27,28]. In this scenario, our results might suggest that mutations causing a moderate loss of CF function lead to less severe changes in epithelial barriers to infection and consequently lower rate of infection with a number of organisms commonly found in the CF lung. Regardless of mechanism, the findings of this study indicate that reduced CFTR function leads to a global disruption of infection control and acquisition.

We have to acknowledge that there are several weaknesses in this study. First, the study was retrospective and patients were enrolled on the basis of being diagnosed with CF and having a sibling diagnosed with CF. This inclusion criterion may have skewed our population as survival of both siblings until entry in our study was required. To assess for a possible bias toward milder CF patients who may have a lower rate of respiratory infection, we compared our data to the general U.S. CF population in the CFF registry and found rates of infection in this study to be similar to the unrelated U.S. CF population (TABLE 2). Another weakness is that we did not apply a correction for age of diagnosis of CF as this event may have been precipitated by a positive respiratory culture. To address this shortcoming, we ensured that subjects had at least one negative respiratory culture for the organism being studied prior to entry into the analysis thereby excluding individuals who were infected at the time of CF diagnosis.

Strengths of this study include sample size, the conservative use of alternative definitions of infection and assessment of lung function prior to the acquisition of the organism. Using a mean survival time of 5 years for the control group ('Minimal' function), 5 years for accrual of all subjects, 10 years of follow-up time and 80% power, we had an adequate sample size to detect a hazard ratio (HR) of 1.3. As shown in FIGURE 1, organisms that demonstrated a significant correlation with CFTR function had HRs that considerably exceeded this threshold. While our main outcome measure was the initial acquisition of an organism, we also stratified patients using 3 other definitions for infection. As highly similar results were obtained when using either of these definitions, we conclude that our results are independent of the criteria used to assign infection status. Inclusion of additional definitions of infection also helped overcome the issue of using a single throat culture (if that was the only culture information for a subject) to determine infection status since throat cultures are known to have poor negative predictive value. By including criteria which require assessment of multiple cultures, correct classification of subjects is more assured. Finally, by assessing lung function prior to the acquisition of the infectious organism analyzed, we were able to evaluate whether the association between predicted CFTR function and infection status was dependent on the severity of lung disease around the time of first infection.

## Conclusions

In conclusion, genotypes predicting 'Minimal' *CFTR *function are associated with increased risk and earlier age of infection by multiple organisms. For those with decreased lung function, 'Residual' *CFTR *genotype no longer provides a protective effect on infection risk. These observations suggest that 1) adjustment for CFTR functional genotype may be necessary in genetic modifier studies of infection, 2) monitoring infection status in patients with mild lung disease, especially children, could be an outcome measure for therapies that augment CFTR function and 3) augmentation of CFTR function may not reduce the risk of infection in patients with severe lung disease.

## List of Abbreviations

*ASP: Aspergillus fumigatus; *ATYP: Atypical mycobacteria; *AX*: *Achromobacter xylosoxidans; BCC*: *Burkholderia cepacia *complex; CF: cystic fibrosis; CFTR: cystic fibrosis transmembrane conductance regulator; CFTSS: U.S. CF Twin and Sibling Study; *EC*: *Escherichia coli; HI*: *Haemophilus influenzae; KP*: *Klebsiella pneumoniae; *M*Pa: *mucoid *Pseudomonas aeruginosa; *MR*Sa*: methicillin resistant *Staphylococcus aureus*; *Pa: Pseudomonas aeruginosa; Sa*: *Staphylococcus aureus; Sm*: *Stenotrophomonas maltophilia*; *Sp*: *Streptococcus pneumoniae; *FirstInfx: first positive culture; ChronInfx: chronic infection: 3 positive cultures within 6 months each 1 month from the previous; MultiInfx: multiple infection: 3 positive cultures in the subjects lifetime; PersInfx: persistently infected: 2 positive cultures each in a separate year within 3 consecutive years; PS: pancreatic sufficient; PI: pancreatic insufficient; FEV_1_: forced expiratory volume in one second; FEV_1_BeforeInfx: maximum CF-specific percentile forced expiratory volume in one second in the year before the first positive culture for an organism

## Competing interests

The authors declare that they have no competing interests.

## Authors' contributions

DMG had full access to all data, contributed to data collection, conception and design of the original idea, data analysis, and drafting of the manuscript. KEM was involved in conception and design of the original idea, and data analysis. SMB contributed to conception and design of the study, and data acquisition. PRS was involved in conception and design of the original study, and data analysis. LBH provided assistance on conception and design of the study, and data analysis. KMN provided input on conception and study design, data acquisition, and administrative, technical and material support. JMC was involved conception and design of the study, data analysis, data acquisition of data, and statistical analysis. GRC was involved in conception and design of the study, data analysis, drafting of the manuscript, and administrative, technical and material support as well as overall study supervision. All authors read, revised and approved the final manuscript.
